# Ultrastructural characters of the spermatozoa in Digeneans of the genus *Lecithochirium* Lühe, 1901 (Digenea, Hemiuridae), parasites of fishes: comparative study of *L. microstomum* and *L. musculus*

**DOI:** 10.1051/parasite/2014050

**Published:** 2014-09-30

**Authors:** Papa Ibnou Ndiaye, Yann Quilichini, Aminata Sène, Vasyl V. Tkach, Cheikh Tidiane Bâ, Bernard Marchand

**Affiliations:** 1 Laboratory of Evolutionary Biology, Ecology and Management of Ecosystems, Faculty of Sciences and Techniques, Cheikh Anta Diop University of Dakar BP 5055 Dakar Senegal; 2 CNRS – University of Corsica, UMR 6134, “Service d’Étude et de Recherche en Microscopie Électronique” 20250 Corte Corsica France; 3 Department of Biology, University of North Dakota 10 Cornell street Grand Forks ND 58202 USA

**Keywords:** Spermatozoon, Ultrastructure, *Lecithochirium microstomum*, *L. musculus*, Hemiuridae, Digenea

## Abstract

This study provides the first ultrastructural data of spermatozoa in the genus *Lecithochirium.* The spermatozoa of *L. microstomum* (from *Trichiurus lepturus* in Senegal) and *L. musculus* (from *Anguilla anguilla* in Corsica) exhibit the general pattern described in the great majority of the Digenea, namely two axonemes with the 9 + “1” pattern typical of the Trepaxonemata, one mitochondrion, a nucleus, parallel cortical microtubules and external ornamentation of the plasma membrane. Spermatozoa of *L. microstomum* and *L. musculus* have some specific features such as the presence of a reduced number of cortical microtubules arranged on only one side of the spermatozoon, the lack of spine-like bodies and expansion of the plasma membrane. The external ornamentation of the plasma membrane entirely covers the anterior extremity of the spermatozoa. The ultrastructure of the posterior extremity of the spermatozoa corresponds to the pattern previously described in the Hemiuridae, characterized by only singlets of the second axoneme. A particularity of these spermatozoa is the organization of the microtubule doublets of the second axoneme around the nucleus in the posterior part of the spermatozoon.

## Abstract (fr)

Cette étude fournit les premières données ultrastructurales sur les spermatozoïdes du genre *Lecithochirium*. Les spermatozoïdes de *L. microstomum* (de *Trichiurus lepturus* du Sénégal) et de *L. musculus* (d’*Anguilla anguilla* de Corse) présentent le modèle généralement décrit chez la grande majorité des digènes, à savoir deux axonèmes de type 9+« 1 » caractéristique des Trepaxonemata, une mitochondrie, un noyau, des microtubules corticaux parallèles et une ornementation externe de la membrane plasmique. Les spermatozoïdes de *L. microstomum* et de *L. musculus* présentent toutefois quelques spécificités telles que la présence d’un nombre réduit de microtubules corticaux disposés d’un seul côté du spermatozoïde, l’absence de corps en forme d’épine et d’expansion de la membrane plasmique. L’ornementation externe de la membrane plasmique recouvre entièrement l’extrémité antérieure des spermatozoïdes. L’ultrastructure de l’extrémité postérieure de ces spermatozoïdes correspond au modèle précédemment décrit chez les Hemiuridae, caractérisée par seulement des singulets provenant des doublets du second axonème. Une particularité de ces spermatozoïdes est l’organisation des doublets de microtubules du deuxième axonème autour du noyau, dans la partie postérieure du spermatozoïde.

## Introduction

Like the majority of hemiuroid digeneans, members of the genus *Lecithochirium* Lühe, 1901 are parasites of the digestive tract of fishes. They belong to the Lecithochiriinae Lühe, 1901, one of the 12 subfamilies of Hemiuridae proposed by Gibson [17]. The systematic position of Lecithochiriinae is problematic. Skrjabin and Guschanskaja [54–59] proposed to remove the Lecithochiriinae from the Hemiuridae. Since, there have been several controversies between authors [5, 10, 16, 18, 19, 32, 61]. However, the Lecithochiriinae are still placed in the Hemiuridae [13, 17].

Ultrastructural data of spermatozoa may provide a useful contribution to understanding of the phylogenetic relationships within the Hemiuridae. In the Platyhelminthes, most ultrastructural features of the spermatozoon proved to be valuable characters for phylogenetic purposes [3, 4, 23–27, 35]. The ultrastructural data, associated with the results of molecular phylogenetic studies, have greatly improved our understanding of the interrelationships in most groups of Platyhelminthes [9, 12, 14, 21, 31, 36–38, 46, 59]. In the Digenea, ultrastructural data of the spermatozoon are available for more than 75 species distributed among 45 families [6, 53]. In the Hemiuroidea, ultrastructural data on spermiogenesis and/or spermatozoa exist for seven species belonging to four families [41]. In the Hemiuridae, such data exist for representatives of only two of the twelve currently recognized subfamilies, namely an elytrophalline, *Lecithocladium excisum* [42], and a hemiurine, *Parahemiurus merus* [41]. The present study is the first work of this kind in the Lecithochiriinae and describes the ultrastructural characteristics of the spermatozoon in two representatives of the genus *Lecithochirium*.

## Material and methods

Adult specimens of *Lecithochirium microstomum* Chandler, 1935 were collected from the digestive tract of *Trichiurus lepturus* (Linnaeus, 1758) (Pisces, Trichiuridae) caught in the Atlantic Ocean, near Dakar (Senegal). Adult specimens of *Lecithochirium musculus* (Looss, 1907) were collected from the digestive tract of *Anguilla anguilla* (Linnaeus, 1758) (Osteichthyes, Anguillidae) caught in Urbino pond, a coastal lagoon of the Mediterranean Sea (Corsica, France).

Live worms were rinsed with a 0.9% NaCl solution and fixed in cold (4 °C) 2.5% glutaraldehyde in a 0.1 M sodium cacodylate buffer at pH 7.2, rinsed in 0.1 M sodium cacodylate buffer at pH 7.2, post-fixed in cold (4 °C) 1% osmium tetroxide in the same buffer for 1 h, rinsed in a 0.1 M sodium cacodylate buffer at pH 7.2, dehydrated in ethanol and propylene oxide, embedded in Spurr’s resin and polymerized at 60 °C for 24 h.

Ultrathin (60–90 nm thick) sections were obtained using an ultramicrotome (Power Tome PC, RMC Boeckeler^®^) with a diamond knife. Sections placed on copper grids were double-stained with uranyl acetate and lead citrate. Sections were placed on gold grids and stained with periodic acid, thiocarbohydrazide and silver proteinate to reveal the presence of glycogen [60].

The grids were examined in a Hitachi H-7650 transmission electron microscope operated at 80 kV, in the “Service d’Étude et de Recherche en Microscopie Électronique” of the University of Corsica (Corte, France).

## Results

**Figures 1–19.**
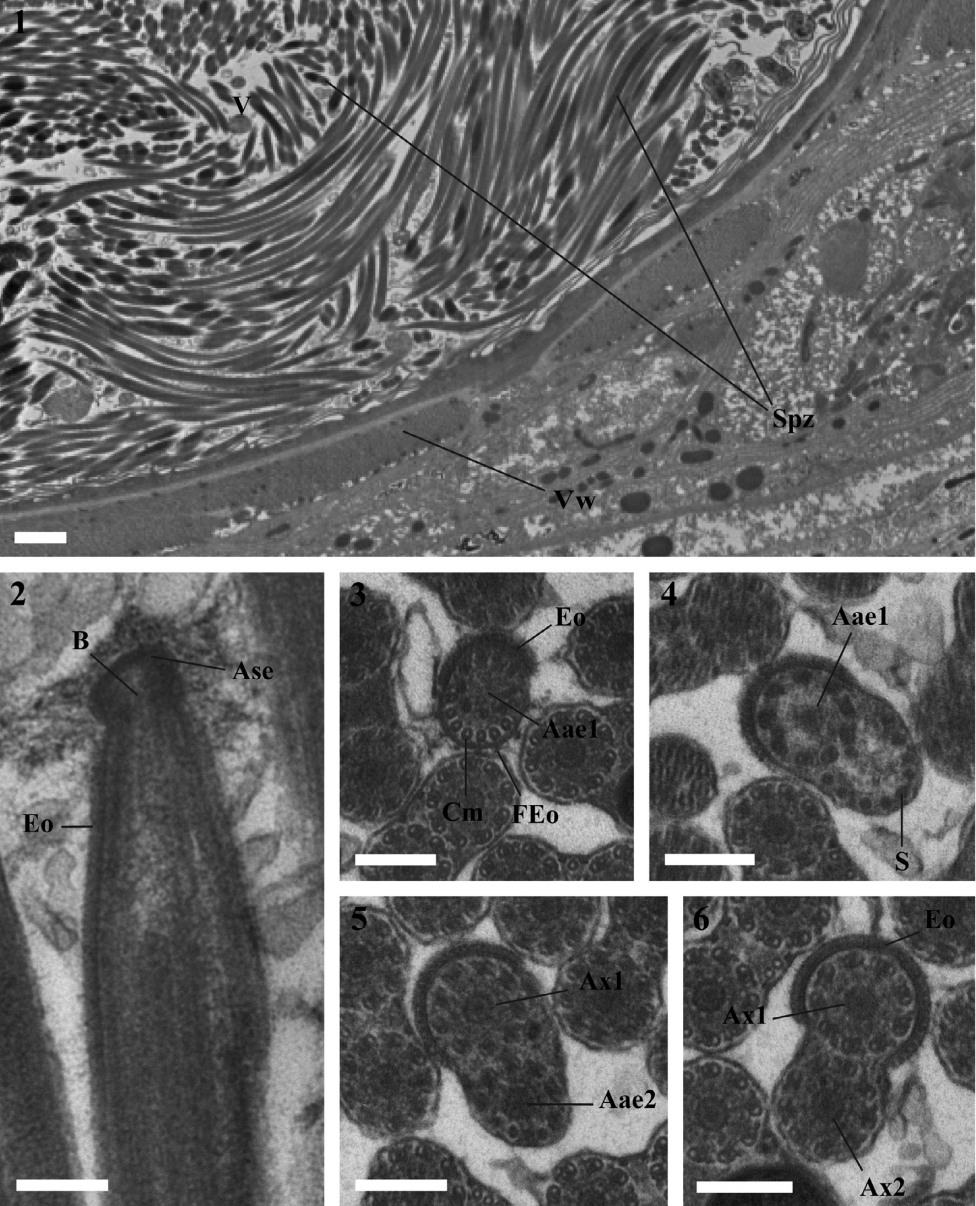

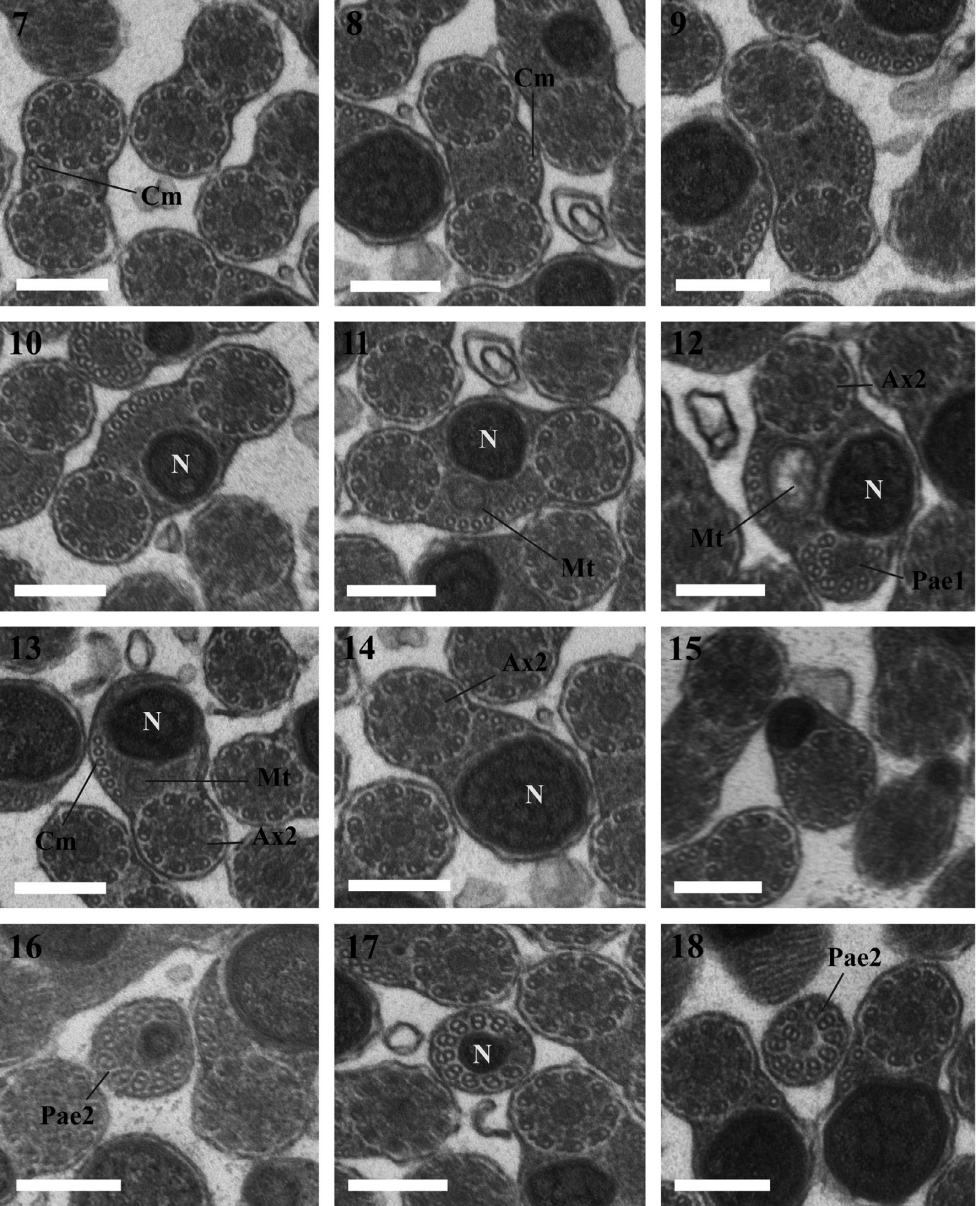

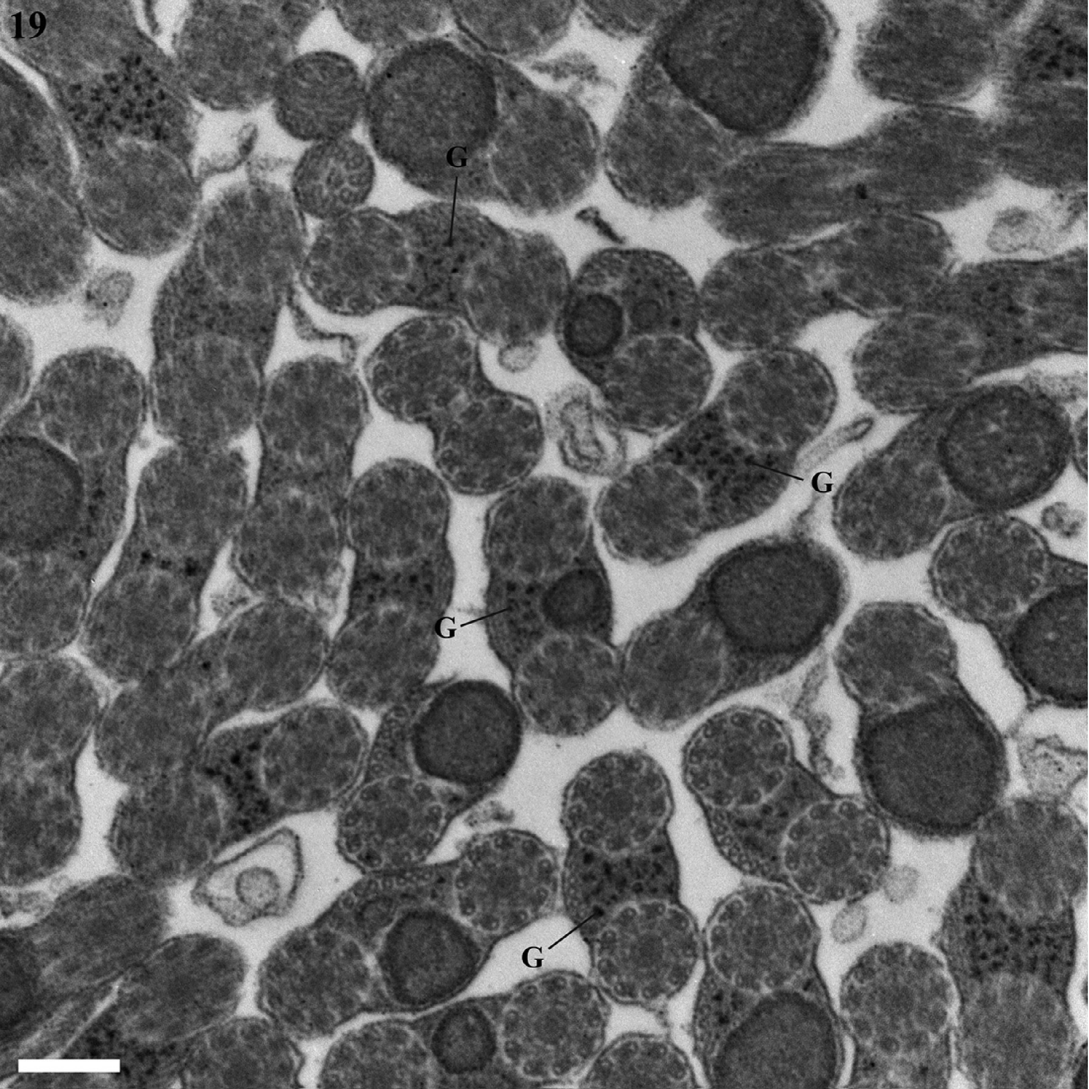
1. A fragment of the seminal vesicle of *Lecithochirium microstomum* containing spermatozoa. Scale bar = 2 μm. Spz = spermatozoon, V = seminal vesicle, Vw = seminal vesicle wall. 2–6. Region I of the spermatozoon of *Lecithochirium microstomum.* Scale bars = 0.2 μm. (2). A longitudinal section of the anterior extremity of the spermatozoon showing the external ornamentation of the plasma membrane. (3). Cross-section in the anterior extremity of the spermatozoon showing the anterior axonemal extremity 1, the external ornamentation of the plasma membrane and some microtubules. (4). Cross-section showing the external ornamentation of the plasma membrane, the anterior axonemal extremity 1 and some singlets of the axoneme 2. (5). Cross-section showing the axoneme 1, the anterior axonemal extremity 2 and the external ornamentation of the plasma membrane. (6). Cross-section with the two axonemes and the external ornamentation of the plasma membrane. Aae1 = anterior extremity of the first axoneme, Aae2 = anterior extremity of the second axoneme, Ase = anterior spermatozoon extremity, Ax1 = first axoneme, Ax2 = second axoneme, B = bulge, Cm = cortical microtubules, Eo = external ornamentation of the plasma membrane, FEo = filamentous ornamentation, S = singlet. 7–12. Cross-sections of Region II of the spermatozoon of *Lecithochirium microstomum.* Scale bars = 0.2 μm. (7–9). Two axonemes and cortical microtubules. (10). Two axonemes, cortical microtubules and the nucleus. (11). Two axonemes, cortical microtubules, the nucleus and the mitochondrion. (12). One axoneme completely formed, nucleus, mitochondrion, cortical microtubules and the disorganization of the first axoneme. Ax2 = second axoneme, Cm = cortical microtubules, Mt = mitochondrion, N = nucleus, Pae1 = posterior extremity of the first axoneme. 13–14. Cross-section of Region III of the spermatozoon of *Lecithochirium microstomum.* Scale bar = 0.2 μm. (13). One axoneme, nucleus, mitochondrion and cortical microtubules. (14). One axoneme, nucleus and cortical microtubules. Ax2 = second axoneme, Cm = cortical microtubules, Mt = mitochondrion, N = nucleus. 15–18. Cross-sections of Region IV of the spermatozoon of *Lecithochirium microstomum*. Scale bars = 0.2 μm. (15). The second axoneme and nucleus. (16). Disorganization of the second axoneme and nucleus. (17). The nucleus surrounded by the doublets of the disorganized axoneme 2. (18). Cross-section in the posterior extremity of the spermatozoon showing only the posterior extremity of the second axoneme. N = nucleus, Pae 2 = posterior extremity of the second axoneme. 19. Transmission electron micrograph of spermatozoa of *Lecithochirium microstomum* showing glycogen granules (G) revealed by the test of Thiéry. Scale bar = 0.2 μm.

Observation of numerous cross- and longitudinal sections of the mature spermatozoa in the seminal vesicle of *Lecithochirium microstomum* and *L. musculus* (Figs. 1 and 20) enabled us to establish the ultrastructural organization of their spermatozoa and distinguish in both species four regions (I–IV) from the anterior to the posterior extremities.

**Figures 20–37.**
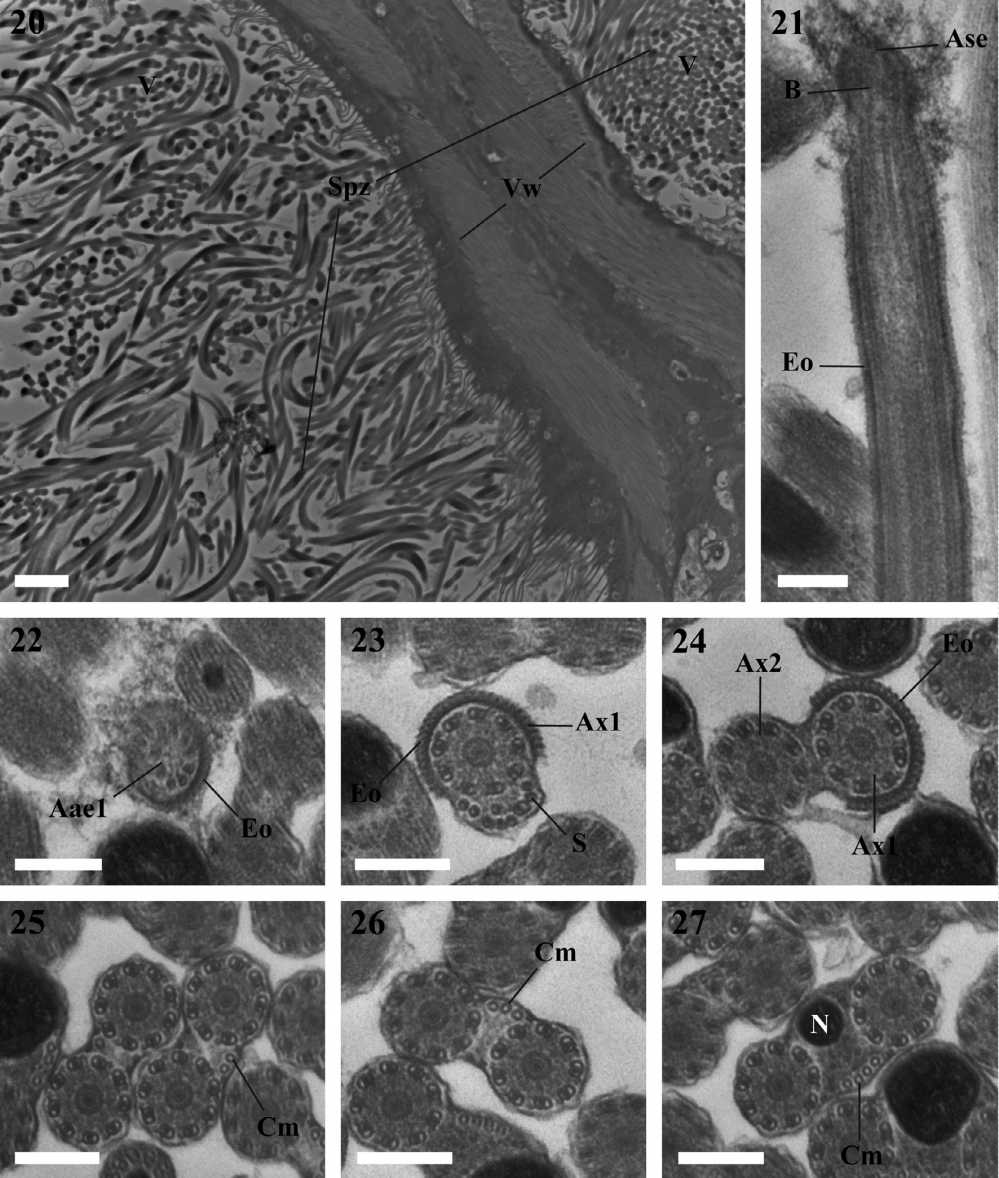

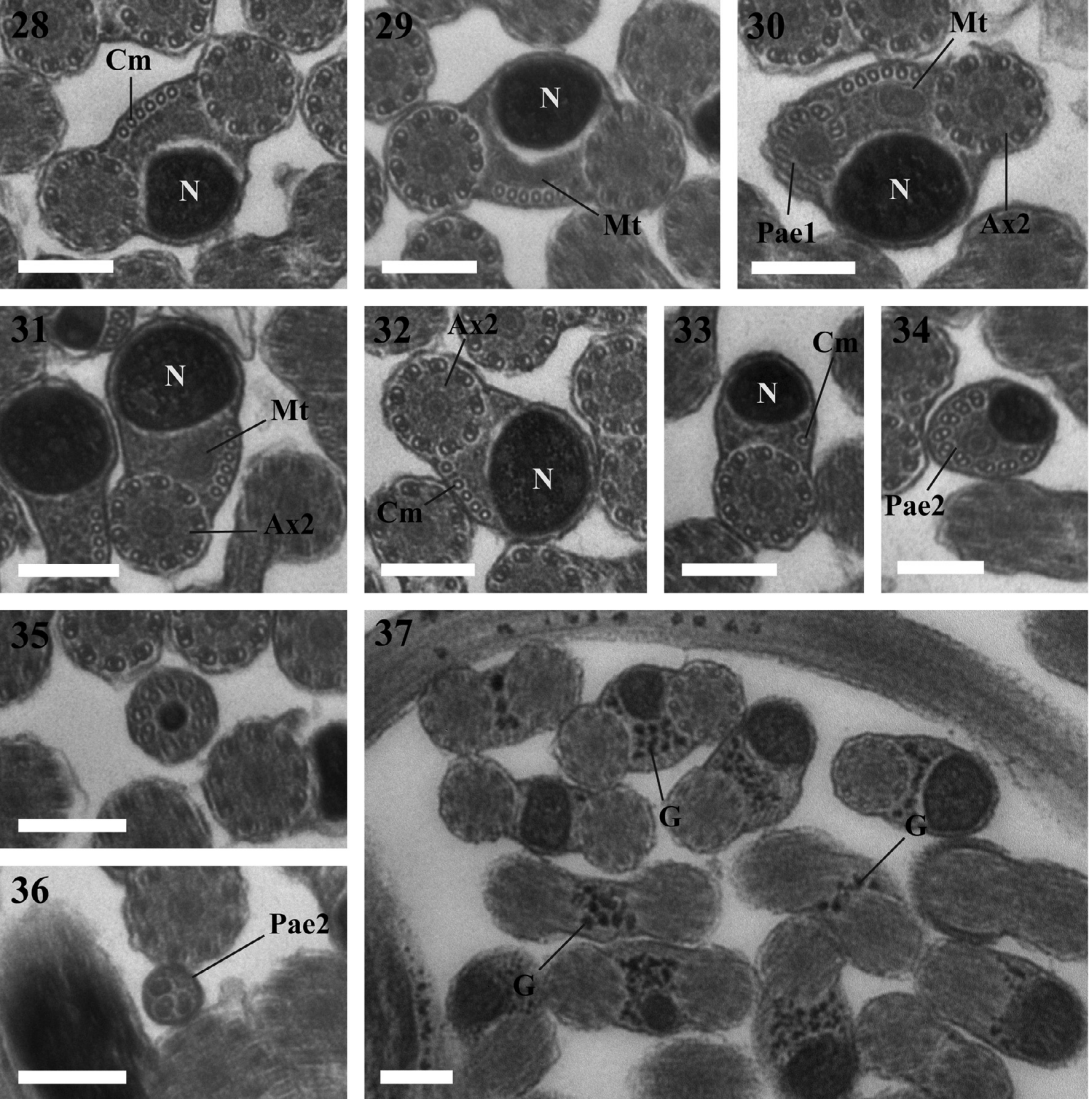
20. A fragment of the seminal vesicle of *Lecithochirium musculus* containing spermatozoa. Scale bar = 2 μm. Spz = spermatozoon, V = seminal vesicle, Vw = seminal vesicle wall. 21–24. Region I of the spermatozoon of *Lecithochirium musculus.* Scale bars = 0.2 μm. (21). A longitudinal section of the anterior extremity of the spermatozoon showing the external ornamentation of the plasma membrane. (22). Cross-section in the anterior extremity of the spermatozoon showing the anterior extremity of the first axoneme and the external ornamentation of the plasma membrane. (23). Cross-section exhibiting the axoneme 1, the external ornamentation of the plasma membrane and singlets of the second axoneme. (24). Cross-section showing the two axonemes and the external ornamentation of the plasma membrane. Aae1 = anterior extremity of the first axoneme, Ase = anterior spermatozoon extremity, Ax1 = first axoneme, Ax2 = second axoneme, B = bulge, Eo = external ornamentation of the plasma membrane, S = singlet. 25–30. Cross-sections of Region II of the spermatozoon of *Lecithochirium musculus.* Scale bars = 0.2 μm. (25–26). Two axonemes and some cortical microtubules. (27–28). Two axonemes, cortical microtubules and the nucleus. (29). Two axonemes, cortical microtubules, the nucleus and the mitochondrion. (30). One axoneme, nucleus, mitochondrion, cortical microtubules and the posterior axonemal extremity 1. Ax2 = second axoneme, Cm = cortical microtubules, Mt = mitochondrion, N = nucleus, Pae1 = posterior extremity of the first axoneme. 31–32. Cross-section of Region III of the spermatozoon of *Lecithochirium musculus.* Scale bars = 0.2 μm. (31). The second axoneme, nucleus, mitochondrion and some cortical microtubules. (32). The second axoneme, nucleus and some microtubules. Ax2 = second axoneme, Cm = cortical microtubules, Mt = mitochondrion, N = nucleus. 33–36. Cross-section of Region IV of the spermatozoon of *Lecithochirium musculus.* Scale bars = 0.2 μm. (33). Cross-section of the spermatozoon showing the second axoneme, nucleus and one cortical microtubule. (34). Cross-section of the spermatozoon showing the second axoneme disorganized and the nucleus. (35). Cross-section of the spermatozoon showing the nucleus surrounded by doublets from the second axoneme. (36). Cross-section showing the posterior extremity of the spermatozoon with only the posterior extremity of the second axoneme. Cm = cortical microtubules, N = nucleus, Pae 2 = posterior extremity of the second axoneme. 37. Transmission electron micrograph of spermatozoa of *Lecithochirium musculus* showing glycogen granules (G) revealed by the test of Thiéry. Scale bar = 0.2 μm.

### Region I (Figs. 2–6, 21–24 and 38I)

This region corresponds to the anterior extremity of the spermatozoon. It exhibits a bulge (Figs. 2 and 21) associated with an external ornamentation of the plasma membrane. Cross-sections in the bulge show the presence of the anterior extremity of the first axoneme, the ornamentation of the plasma membrane and some cortical microtubules under the plasma membrane associated with filamentous ornamentation (Fig. 3). Soon the first axoneme appears completely formed and the singlets of the second axoneme. The appearance of the singlets of the second axoneme coincides with the disappearance of the cortical microtubules (Figs. 4 and 23). The posterior part of this region exhibits only two axonemes and the external ornamentation of the plasma membrane located only around the first axoneme (Figs. 5, 6 and 24).

**Figure 38.**
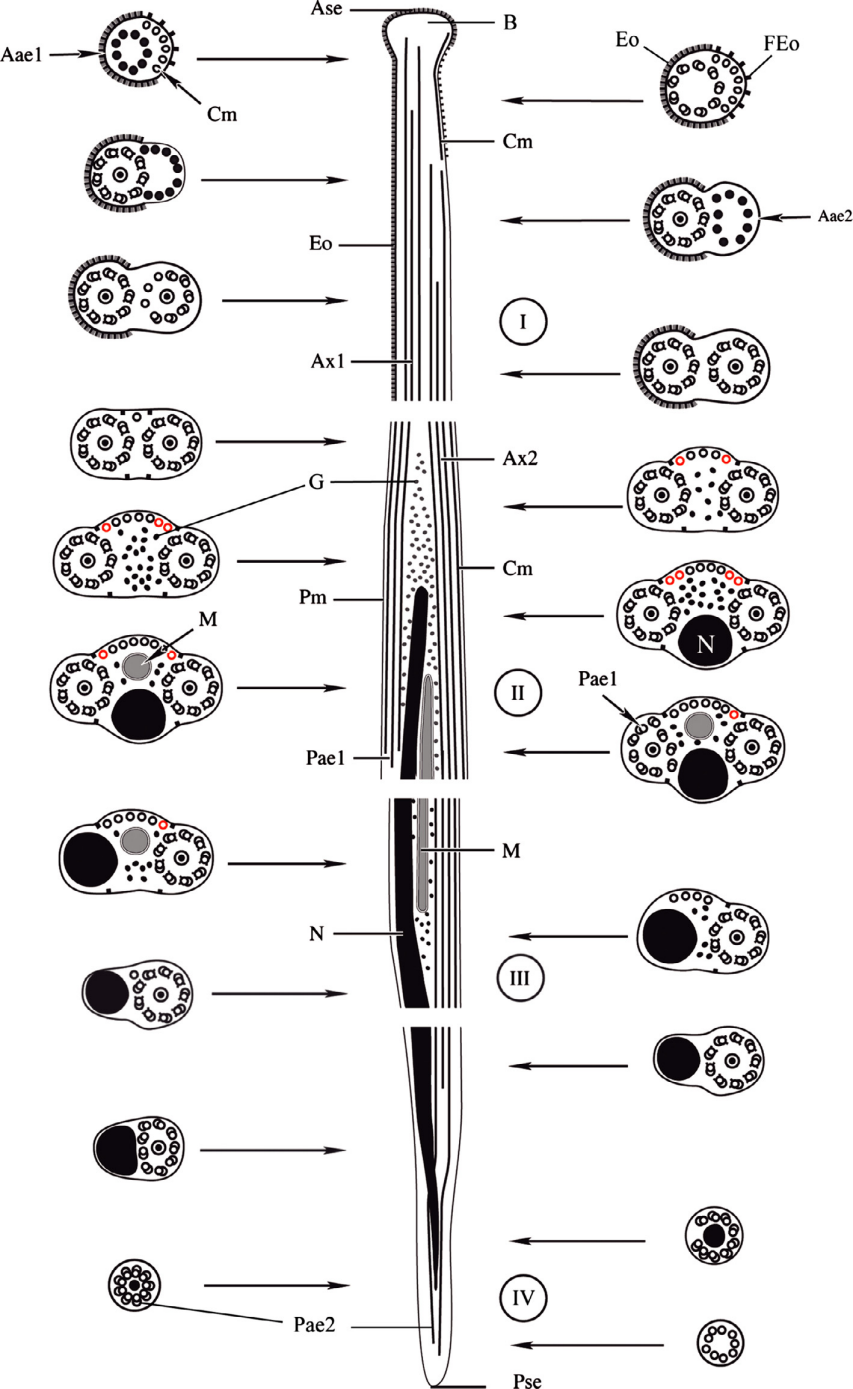
Schematic reconstruction of the spermatozoon in the genus *Lecithochirium*: *L. musculus* (in black), *L. microstomum* (in black + red). Aae1 = anterior extremity of the first axoneme, Aae2 = anterior extremity of the second axoneme, Ase = anterior spermatozoon extremity, Ax1 = first axoneme, Ax2 = second axoneme, B = bulge, Cm = cortical microtubules, Eo = external ornamentation of the plasma membrane, FEo = filamentous ornamentation, G = granules of glycogen, M = mitochondrion, N = nucleus, Pae1 = posterior extremity of the first axoneme, Pae2 = posterior extremity of the second axoneme, Pm = plasma membrane, Pse = posterior spermatozoon extremity.

### Region II (Figs. 7–12, 25–30 and 38II)

This region is characterized by the disappearance of the external ornamentation of the plasma membrane (Figs. 7 and 25), the gradual appearance of cortical microtubules disposed on only one side of the spermatozoon (Figs. 7–9, 25, 26 and 30) and the appearance of the nucleus (Figs. 10–11 and 27) and the mitochondrion (Figs. 11, 12, 29 and 30) in the posterior part of this region. In *L. microstomum* the number of cortical microtubules increases in this region by one (Fig. 7) to eight (Figs. 9–11). In *L. musculus*, this number increases by one (Fig. 25) to six in association with the nucleus (Figs. 28–30). Cross-sections in the posterior extremity of this region show the posterior extremity of the first axoneme in both species (Figs. 12 and 30).

### Region III (Figs. 13–14, 31–33 and 38III)

This region is characterized by the disappearance of the first axoneme in both *L. microstomum* and *L. musculus.* The number of cortical microtubules in *L. microstomum* decreases from 6 (Fig. 13) to 3 (Fig. 14). In *L. musculus*, this number decreases to 5 (Figs. 31–32) and to 1 (Fig. 33). This region is also characterized by the disappearance of the mitochondrion (Figs. 13–14 and 31–33).

### Region IV (Figs. 15–18, 33–36 and 38IV)

This region corresponds to the posterior region of the spermatozoon. Cortical microtubules disappear and the nucleus size decreases progressively (Figs. 15–17, 34 and 35). Cross-sections in the posterior part of the spermatozoon show the reduced nucleus surrounded by doublets of the disorganized second axoneme (Figs. 17 and 35). The posterior extremity of the spermatozoon is characterized by the disappearance of the nucleus and the presence of only the posterior extremity of the axoneme 2 (Figs. 18 and 36).

The micrographs in Figures 19 and 37 show a positive result of the Thiéry test, exhibiting a reduced presence of glycogen granules along the mature spermatozoa in both species studied.

## Discussion

Spermatozoa of *L. microstomum* and *L. musculus* exhibit the general pattern described in most of the Digenea, namely two axonemes of the 9 + “1” pattern of trepaxonematans [15], a nucleus, one mitochondrion and parallel cortical microtubules, and are tapered at both ends [8, 11, 22, 23, 33, 39, 41, 52, 53]. However, spermatozoa of *Lecithochirium* are also characterized by several specific features.

### The anterior spermatozoon extremity

The spermatozoon extremity of the two species is filiform and exhibits a bulge described here for the first time in the Digenea. It is also covered by an external ornamentation of the plasma membrane. In the other two Hemiuridae studied so far, namely *Lecithocladium excisum* and *Parahemiurus merus* [41, 42], the anterior extremity of the spermatozoon has only one axoneme and external ornamentation of the plasma membrane. The unique feature of the spermatozoa of *Lecithochirium* is the presence of a few short cortical microtubules (6) in the anterior extremity of the spermatozoon in addition to the filamentous external ornamentation of the plasma membrane. In the Hemiuroidea, this type of anterior spermatozoon extremity was previously described only in *Gonapodasmius* sp. by Justine and Mattei [28, 30] (see Table 1) and in *Prosorchis palinurichthi* [45]. In 2011, Quilichini et al. [48] distinguished three types of digenean spermatozoa according to the localization of the external ornamentation: type 1 (external ornamentation in the anterior extremity of the spermatozoon), type 2 (external ornamentation at a more posterior level) and type 3 (absence of external ornamentation). According to this criterion, the hemiuroidean spermatozoa can be classified as the first type (Table 1).

**Table 1.** Spermatological characters in the superfamily of Hemiuroidea (Ndiaye et al. [41] completed).

Families and species	Spermatological characters								References
	Ase	Eo			Cm		M	Pse	
		+/−	Loc	Eo/Cm	Bund	*n*			
Didymozoidae									
*Didymocystis wedli*	?	−	NA	NA	–	0	1	?	[47]
*Didymozoon* sp.	?	−	NA	NA	−	0	1	?	[29]
*Gonapodasmius* sp.	1Ax-Eo-Cm	+	RAnt	+	2	36	1	2F	[28, 30]
Hemiuridae									
*Lecithocladium excisum*	1Ax-Eo	+	RAnt	−	1	8	1	1Ax	[42]
*Parahemiurus merus*	1Ax-Eo		RAnt	−	1	5	1	1Ax	[41]
*Lecithochirium microstomum*	1Ax-Eo-Cm	+	RAnt	+	1	8	1	1Ax	Present study
*Lecithochirium musculus*	1Ax-Eo-FEo-Cm	+	RAnt	+	1	6	1	1Ax	Present study
Lecithasteridae									
*Aponurus laguncula*	1Ax-Eo	+	RAnt	−	1	10	1	D+S	[49]
Sclerodistomidae									
*Prosorchis ghanensis*	?	?	NA	NA	1–2	13–15	1	?	[25]
*Prosorchis palinurichthi*	1Ax-Cm	+	RAnt	+	1	25	1	1Ax	[45]

Spermatozoon characters: Ase, anterior spermatozoon extremity; Ax, axoneme; bund, number of bundles of cortical microtubules; Cm, cortical microtubules; D, doublets; Eo, external ornamentations of the plasma membrane; FEo, filamentous ornamentation of the plasma membrane; Eo/Cm, association of external ornamentations and cortical microtubules; F, flagellum; loc, location of external ornamentations; *n*, maximum number of cortical microtubules; NA, not applicable; Pse, posterior spermatozoon extremity; Rant, anterior region; +/–, presence/absence; ?, unknown data.

In the majority of digeneans studied up to now, the external ornamentation of the plasma membrane is associated with spine-like bodies [7, 39, 41, 43, 51]. However, these structures are absent in the spermatozoon of all studied Hemiuroidea.

### Cortical microtubules

Cortical microtubules were described in spermatozoa of almost all of the digeneans studied so far. They are generally disposed in two bundles. One of the specific features of the spermatozoon of the Hemiuridae is the presence of only one reduced bundle of cortical microtubules on one side of the spermatozoon [41, 42 and present study]. Up to now, the maximum number of cortical microtubules encountered in Hemiuridae was eight in *Lecithochirium excisum* [42] and *Lecithochirium microstomum* (this study). The spermatozoon of *Lecithochirium musculus* exhibits only six cortical microtubules (this study). *P. merus* [41] has the smallest number of cortical microtubules (5) in Hemiuridae. The number of cortical microtubules is higher in other Hemiuroidea: 10 in *Aponurus laguncula* [49], 13 in *Prosorchis ghanensis* [25], 25 in *Prosorchis palinurichthi* [45] and 36 in *Gonapodasmius* sp. [28, 30].

The principal difference between *L. microstomum* and *L. musculus* is in the number of cortical microtubules. In *L. microstomum* the maximum number of cortical microtubules is observed in the region of the spermatozoon with only the two axonemes, glycogen granules and nucleus. However, in *L. musculus*, the maximum number of cortical microtubules is six and is situated in a region of the spermatozoon with the simultaneous presence of the nucleus and the mitochondrion.

### Mitochondrion

Similar to other taxa of the Hemiuroidea studied up to now (Table 1), the spermatozoa of *L. microstomum* and *L. musculus* have only one mitochondrion. Among more than 75 studied species of Digenea, spermatozoa with one mitochondrion were described in 40 species (see [6] completed by [40, 41, 45]). In the remaining species, spermatozoa with two or three mitochondria were described [1, 2, 20, 34, 44, 51].

### Posterior spermatozoon extremity

The posterior part of the spermatozoon is identical in both species of *Lecithochirium* examined in our study and characterized by the presence of only the nucleus and the posterior extremity of the second axoneme. The posterior extremity of the spermatozoa is particular and characterized by a disorganization of the axoneme associated with the migration of the posterior extremity of the nucleus in the central part of the disorganized axoneme. Thus, in the end of this posterior extremity, the nucleus disappears, and instead only the axonemal singlets are present in the posterior end of the spermatozoon. A posterior end of the spermatozoon with only axonemal singlets was described in all the Hemiuridae studied so far [41, 42]. This suggests that this character can be a useful criterion for phylogenetic purposes. The presence of the nucleus in the posterior part of the spermatozoon was also described in all studied hemiurids [41, 42, present study]. In *P. merus*, some cortical microtubules are described in the posterior extremity of the spermatozoon. However, in *L. microstomum* and *L. musculus* (present study), cortical microtubules in the posterior extremity of the spermatozoon are absent. Similar to the other Hemiuridae studied to date [41, 42], the spermatozoon in *Lecithochirium* presents the type III or Cryptogonimidean type characterized by the absence of cortical microtubules, and the sequence posterior extremity of the nucleus then posterior extremity of the second axoneme [50]. The terminal character is coincident in all Hemiuridae. However, the end of the cortical microtubules is different in *Lecithocladium.*

### Glycogen

The presence of glycogen was described in most of the spermatozoa of digeneans described up to now. However, the particularity of the Hemiuridae is the presence of a reduced quantity of glycogen. We believe that it is necessary to perform more studies of this character to clarify its potential importance for the phylogeny of the Hemiuridae.
